# Integrative comparative analysis of avian chromosome evolution by *in-silico* mapping of the gene ontology of homologous synteny blocks and evolutionary breakpoint regions

**DOI:** 10.1007/s10709-023-00185-x

**Published:** 2023-03-20

**Authors:** Jules Claeys, Michael N. Romanov, Darren K. Griffin

**Affiliations:** 1 School of Biosciences, University of Kent, Canterbury, CT2 7NJ UK; 2 L. K. Ernst Federal Research Centre for Animal Husbandry, Dubrovitsy, Podolsk, Moscow Oblast 142132 Russia; 3 The Sainsbury Laboratory, Norwich Research Park, Norwich, NR4 7UH UK

**Keywords:** Avian genome, Chromosome evolution, Evolutionary breakpoint regions, Multispecies homologous synteny blocks, Gene ontology, Bioinformatics tools

## Abstract

Avian chromosomes undergo more intra- than interchromosomal rearrangements, which either induce or are associated with genome variations among birds. Evolving from a common ancestor with a karyotype not dissimilar from modern chicken, two evolutionary elements characterize evolutionary change: homologous synteny blocks (HSBs) constitute common conserved parts at the sequence level, while evolutionary breakpoint regions (EBRs) occur between HSBs, defining the points where rearrangement occurred. Understanding the link between the structural organization and functionality of HSBs and EBRs provides insight into the mechanistic basis of chromosomal change. Previously, we identified gene ontology (GO) terms associated with both; however, here we revisit our analyses in light of newly developed bioinformatic algorithms and the chicken genome assembly galGal6. We aligned genomes available for six birds and one lizard species, identifying 630 HSBs and 19 EBRs. We demonstrate that HSBs hold vast functionality expressed by GO terms that have been largely conserved through evolution. Particularly, we found that genes within microchromosomal HSBs had specific functionalities relevant to neurons, RNA, cellular transport and embryonic development, and other associations. Our findings suggest that microchromosomes may have conserved throughout evolution due to the specificity of GO terms within their HSBs. The detected EBRs included those found in the genome of the anole lizard, meaning they were shared by all saurian descendants, with others being unique to avian lineages. Our estimate of gene richness in HSBs supported the fact that microchromosomes contain twice as many genes as macrochromosomes.

## Introduction

In comparative and evolutionary genomics (e.g., Hardison 2003; Jarvis et al. 2014; Itan et al. 2016), research is focused on comparing the structure and function of the genomes of different species, thereby providing insights into their evolution (e.g., Zhang et al. 2014; Griffin et al. 2015; O’Connor et al. 2018a). Chromosomes undergo rearrangements during evolution (Rogers 2015), including fissions, fusions, deletions, inversions, translocations, and duplications. In addition to well-established cytogenetic methods such as fluorescence in situ hybridization (FISH), bioinformatic tools are now frequently used to analyze and compare chromosomes from various species and identify these chromosomal rearrangements (e.g., Romanov et al. 2005; Modi et al. 2009; Schmid et al. 2015; Kretschmer et al. 2021). This has resulted in the discovery and analysis of homologous synteny blocks (HSBs) and evolutionary breakpoint regions (EBRs) (Larkin et al. 2009; Damas et al. 2018). HSBs are shared by various species and exhibit a common evolution from a single ancestor. On the other hand, EBRs that can be re-used in the genome evolution delineate HSBs and are found in the places where chromosomes break and then rejoin (Sankoff 2009; Griffin et al. 2015; O’Connor et al. 2018a).

HSBs and EBRs constitute important genomic regions that may provide insights into the evolution of the genome and the species to which they belong. The chromosomes of avian species have been examined through sequence-based comparison in Farré et al. (2016). This resulted in the discovery of 1021 EBRs, many of which were lineage-specific. Five sets of multispecies homologous synteny blocks (msHSBs) were created and utilized for hypothesized ancestral genomes of birds, archosaurians, archosaurians/testudines, sauropsids, and amniotes. A total of 1746 msHSBs, or 76.3% of the chicken genome, was found in birds. The fact that the msHSBs exceed the maximum predicted length suggests that they may have survived during the evolution of the genomes of birds and reptiles (Farré et al. 2016).

Previously, features of genomic organization in birds, including macro- and microchromosomes, their rearrangements, HSBs and EBRs, were the subject of our studies using the chicken genome assembly as a reference (e.g., Romanov et al. 2014a,b; Lithgow et al. 2014; Damas et al. 2018; O’Connor et al. 2018b). Datasets from 21 avian genomes and one outgroup of reptile species were uploaded into a chromosomal browser called Evolution Highway (Murphy et al. 2005; Romanov et al. 2014b). Using FISH, we rebuilt scaffold-based assemblies, and analysis of those showed a more sophisticated rearrangement pattern, including changes in microchromosomes. The chicken and zebra finch were also evaluated for the presence of EBRs in relation to regional recombination rate, although the findings were not significant (Romanov et al. 2014b).

Using Evolution Highway and BioMart databases (Kasprzyk 2011), Romanov et al. (2014b) attempted to uncover more information about the function of these EBRs, and Farré et al. (2016) reported the presence of EBRs and the taxa to which they are related to. O’Connor et al. (2018a) sought to map the structure of the diapsid common ancestor genome to learn more about these genetic elements. The 397 msHSBs and the respective EBRs were visualized based on the genome sequence alignment.

In gene ontology (GO) research (Ashburner et al. 2000; The Gene Ontology Consortium 2019), the genomes and gene databases of several species are combined that use a standard vocabulary to characterize the suites of properties of genes and their products. Consequently, the GO databases are established to analyze and annotate functionally the gene content of a genome or a genomic region of interest such as HSBs and EBRs, although in our previous study (Romanov et al. 2014b) we were unable to infer significant and meaningful GO results for these regions in birds. Since then, we have efficiently improved algorithms in our bioinformatic pipeline (O’Connor et al. 2018a), and essentially updated and improved versions of the chicken reference assembly and BioMart/GO databases have been released. Collectively, these bioinformatic improvements suggested to revisit and re-analyze the previous data (Romanov et al. 2014b).

In this regard, we re-analyzed the msHSB and EBR data for better assembled bird genomes in light of improved bioinformatic algorithms and recent genomic sequence and database updates. Therefore, the current investigation aimed to look *in silico* at the distribution, quantity, and GO of genes found in avian msHSBs and EBRs. This has revealed information on the function of genes in msHSBs by determining whether or not functionally related sets of genes on the same chromosome have been preserved during evolution. In terms of EBRs, the objective was to learn if the function of genes associated to EBRs can be also relevant to the evolution of bird species. This study deepens our understanding of how the localization and function of msHSBs and EBRs relate to avian evolution.

## Materials and methods

### Genomes

The genomes of six different species of birds and a species of lizard were used to reconstruct the msHSBs as well as the EBRs of ancient birds and an avian/dinosaur ancestor. Chicken (*Gallus gallus*; GGA) was selected as a reference genome (International Chicken Genome Sequencing Consortium 2004) and compared to the genomes of zebra finch (*Taeniopygia guttata*; Warren et al. 2010), turkey (*Meleagris gallopavo*; Dalloul et al. 2010), Pekin duck (*Anas platyrhynchos*; Huang et al. 2013), budgerigar (*Melopsittacus undulatus*; Ganapathy et al. 2014) and ostrich (*Struthio camelus*; Zhang G. et al. 2014; Zhang J. et al. 2015).

**Fig. 1:**
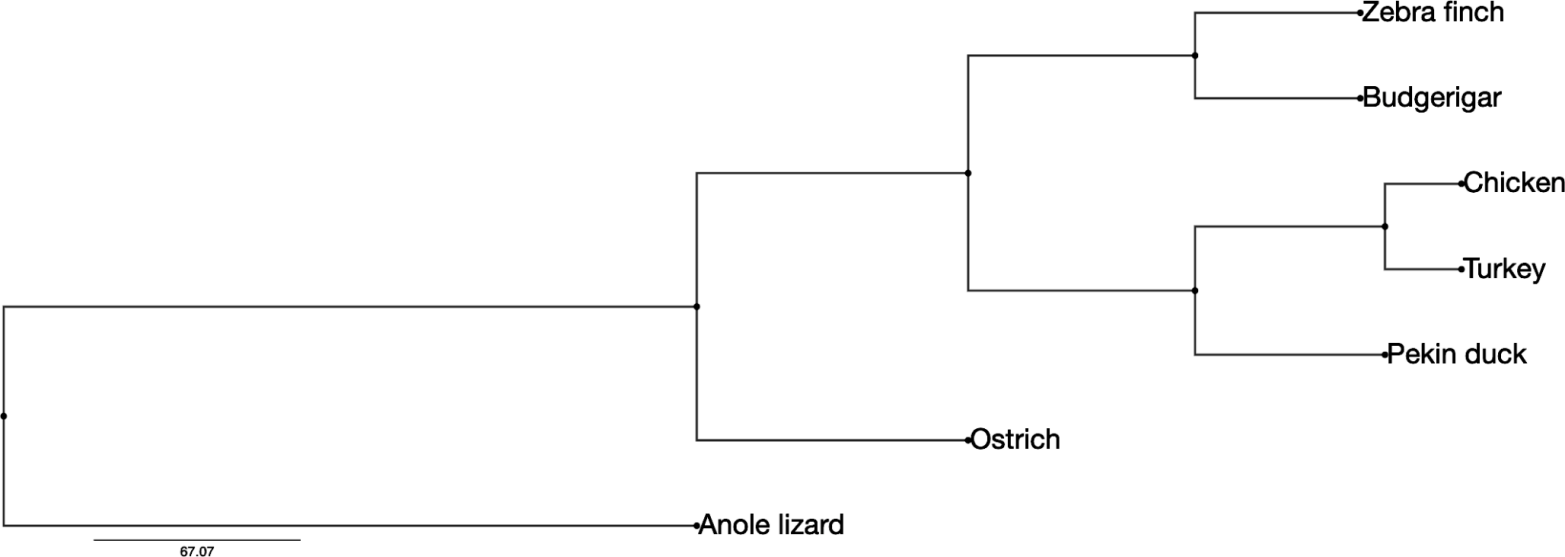
Cladogram of avian lineages for the six species studied, with the lizard used as an outgroup. The tree was visualized using the Phylo.io web application (Robinson et al. 2016) and scaled in million years ago. The respective Newick format tree can be written as ((((Zebra finch,Budgerigar),((Chicken,Turkey),Pekin duck)),Ostrich),Anole lizard);

These six species are all part of the class Aves but belong to different orders in most cases, or to different genera for the chicken and the turkey (Fig. 1). Both the turkey and chicken are members of order Galliformes, but the turkey is part of the *Meleagris* genus while the chicken is a member of the *Gallus* genus. The next closest species to the Galliformes is the duck as a member of the Anseriformes, which is in the same superorder as the Galliformes, the Galloanserae. At the infraclass level, both the zebra finch and the budgerigar are part of the same infraclass Neognathae as the chicken but are both in the Neoaves superorder (Maddison and Schulz 2007). The zebra finch is part of the Passeriformes order, and the budgerigar belongs to the Psittaciformes. The species most distantly related to the five others is the ostrich, in the Struthioniformes order, which falls within the distinct infraclass of the palaeognaths. We also used the green anole (*Anolis carolinensis*) lizard genome (Alföldi et al. 2011) as an outgroup to identify EBRs in the avian evolution.

### Bioinformatic tools and a pipeline to study msHSBs, EBRs, and their GO

Several bioinformatic tools and algorithms were used to carry out this study and formed an inhouse msHSB/EBR mining pipeline (O’Connor et al. 2018a; Abdelmanova et al. 2021). The latter implied the consequent use of the following components and applications: Evolution Highway → LiftOver → BioMart → DAVID.

#### Evolution highway

**Fig. 2:**
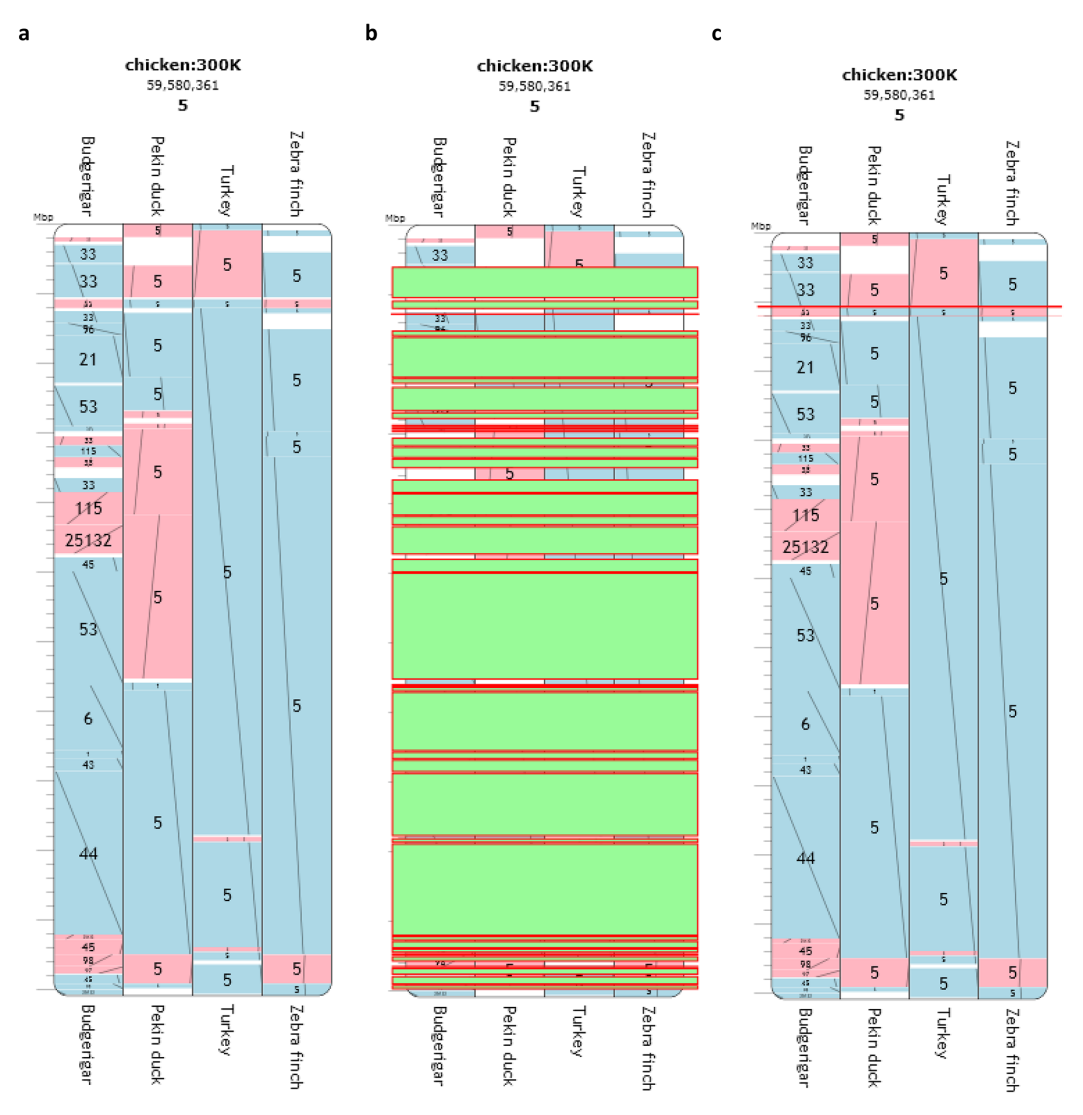
Examples of representation of chicken chromosome 5 and the appropriate aligned chromosomes of multiple bird species using the Evolution Highway genome browser: **a** with pairwise HSBs shown in blue and red, **b** with their multispecies HSBs shown in green, and **c** with a single EBR represented as a red line. Chicken:300 K denotes the 300-Kb resolution window

This genome browser visually represents the comparison of the genomes of multiple amniote species aligned to the genome of a reference species (see an example in Fig. 2). The web tool makes it possible to identify and characterize msHSBs, EBRs, their localization, including their start and end positions (in bp), and their length (Murphy et al. 2005; Romanov et al. 2014b). Evolution Highway was previously used for studying many avian species (e.g., Romanov et al. 2014b; Farré et al. 2016; O’Connor et al. 2018a,b; Kiazim et al. 2021). As aforementioned, we used the chicken genome as the reference, applied it to the total set of chromosomes available in the genomes of zebra finch, Pekin duck, turkey, budgerigar and ostrich, and aligned with them at the 300-Kb resolution. The msHSBs and EBRs were classified as such using Evolution Highway, if they occurred in, and were shared by, all the species compared. We examined the output chromosome diagrams (see examples in Fig. 2) that represented alignments of genome sequences of the above birds identified against the reference chicken genome (Romanov et al. 2014b). Using Evolution Highway, this approach resulted in lists of 649 msHSBs and 21 EBRs including all the relevant information. For EBRs we also did an alignment by adding the genome of the anole lizard to look at EBRs specific to bird lineages.

#### LiftOver

Being a part of the UCSC Genome Browser project (Hinrichs et al. 2010), the LiftOver (or Lift Genome Annotations) web tool converts coordinates from one genome assembly to another. This conversion follows a certain percentage of match between the two genomes and removes all extra conversions, which could have been obtained from a single msHSB or EBR. Once the lists of msHSBs and EBRs were generated, the msHSBs coordinates were converted from the chicken genome assembly galGal4 (an older version used by Evolution Highway; Schmid et al. 2015) into galGal6 (GRCg6a 2018), a more recent assembly version. When running LiftOver, only those msHSBs that had a percentage of match between the two genomes over 90% were kept. As a result, 630 distinct conversions of msHSBs were obtained. For EBRs, a lower match of 24% was manually selected, which at the end gave 19 EBRs.

#### BioMart

The BioMart Ensembl Genes Database version 95 that contained information of curated and annotated genes for various organisms (Kasprzyk 2011; Zerbino et al. 2018) and its dataset of chicken genes from galGal6 were used to determine the location of msHSBs and EBRs. We then produced a list composed of the chicken genes present within each region of interest and on both DNA strands. BioMart also provided information about orthologous human genes best annotated among all sequenced genomes, including gene stable ID and name, chromosome name, human-chicken orthology type, and confidence score. This generated two lists of genes filtered by orthology type and confidence score: one for the genes present in msHSBs, and the other one for the those present in EBRs as well as the respective information about them. For both studies, these lists were further filtered to keep only the genes whose %ID for target gene identical to query gene, and for query gene identical to target gene, was higher than 70%, as it was experimentally tested and effectively implemented in a previous study (O’Connor et al. 2018a).

#### DAVID

The final online tool employed in this study was DAVID, or Database for Annotation, Visualization and Integrated Discovery (Huang et al. 2009a, b; DAVID Knowledgebase 6.8). Using Ensembl Gene IDs as the gene list identifiers, this tool compared GO of an obtained experimental gene list against a background gene list. This procedure produced a GO term enrichment analysis in the form of gene–GO term functional annotation charts and functional annotation clusters for both msHSBs and EBRs. Theoretically, background gene lists could be, for example, a total set of all human genes or those ones that corresponded to certain chromosomes. O’Connor et al. (2018a) experimentally tested both sets of human orthologs and determined that the background GO analysis list should only include results for those chicken chromosomes where the msHSBs and EBRs were found. Following here the same approach, we determined GO enrichment clusters and single GO terms in genomic regions of interest (i.e., msHSBs or EBRs) or in whole chromosomes. In particular, the obtained charts gave information on GO terms, the number of genes and their names in a GO term, their statistical relevance through their *p*-value, and the percentage of the genes on the list that were included in a GO term. The GO clusters regrouped terms having a similar biological meaning as they shared similar gene members. The clusters also provided the genes present in each term, their statistical relevance, and an enrichment score for a cluster to rank their significance. As statistical values, an enrichment score of ≥ 1.3 and a *p*-value < 0.05 were used for significant clusters, while a *p*-value < 0.05 was considered for significant GO terms. Additionally, a false discovery rate (FDR) threshold of 5% was used to establish final numbers of significant clusters and GO terms.

## Results

Using the Evolution Highway genome browser (Fig. 2) and LiftOver, we identified the 630 msHSBs and 19 EBRs used further for deriving BioMart lists of genes. BioMart also generated two lists of background genes, one for the msHSBs and one for the EBRs. As the genes from the msHSBs genes list were only present on chromosomes GGA1 to GGA15, GGA17 to GGA24, GGA26 to GGA28, and GGAZ, the corresponding background gene list was composed of all human orthologous genes on the above 27 chicken chromosomes. For the EBRs background list, genes from GGA4 to GGA6, GGA8, GGA9, GGA14, and GGA18 were respectively used. The two background gene lists obtained were then used to run DAVID.

### msHSBs

As a result of the Evolution Highway-based analysis (Fig. 2b), the 630 msHSBs had a total length of 807,632,434 bp that represented about 2/3 of the chicken genome (~ 1.2 Gb). Using BioMart, we derived orthologous human genes, and after their initial filtering by orthology type and confidence score 7896 genes were identified for further analysis. This corresponded to a gene richness of 12.5 filtered genes per 1 msHSB. Average gene density was 9.8 genes per 1 Mb across all msHSBs.

After subsequent filtering by %ID, we compared the gene content in microchromosomes and macrochromosomes. Within 482 msHSBs in macrochromosomes, a total of 3796 orthologous human genes were found, meaning about 7.9 genes per 1 msHSB in macrochromosomes. In microchromosomes, 1179 genes were present in 148 msHSBs, which implied around 8 genes per 1 msHSB, suggesting that the gene richness was similar in microchromosome- and macrochromosome-specific msHSBs. However, gene density differed in msHSBs of macro- and microchromosomes. In macrochromosomes, msHSBs made up for 691,169,434 bp, in which 3796 orthologous human genes occurred. This meant a gene density of 5.5 genes per 1 Mb in macrochromosomal msHSBs. In microchromosomes, msHSBs made up for 116,463,000 bp and 1179 genes were present in them, with gene density being 10.1 genes per 1 Mb.

Due to the high number of genes present in macrochromosomes, only their single GO terms were studied (Table 1). Seventy-nine single GO terms, with the largest one being linked to “Phosphoprotein“, were found making up for 2046 genes. In microchromosomes, the single GO term “Phosphoprotein“ was also found for 672 genes, suggesting that across all msHSBs we had 2718 genes whose functionality was linked to “Phosphoprotein.”

In microchromosomes, the following GO clusters and terms for separate microchromosomes were retrieved (Table 1):

- GGA11: one significant annotation cluster was found but it did not pass the FDR test, meaning that this cluster should not be considered significant. The respective functional annotation chart did not reveal any significant GO terms.
- GGA12: neither any cluster passed the FDR test, nor any significant single GO terms was discovered. Similarly, the functional annotation chart and functional annotation clustering did not reveal any significant single GO term or cluster on GGA18, GGA22 and GGA28.
- GGA13: a single significant cluster and 19 significant single GO terms were identified. The cluster included GO terms for neuroactive ligand-receptor interaction, postsynaptic cell membrane, ion transmembrane transport, and synapse. Nineteen significant single GO terms showed the same functionalities and, additionally, protein binding.
- GGA14: we only showed single GO terms. These included such functionality as phosphate binding and interaction with TP53.
- GGA15: one significant cluster was displayed that passed the FDR test. This cluster included the term TPR repeat. In addition, the functional annotation chart found significant terms for acetylation, protein complex, RING and polysome.
- GGA19 showed two clusters, but they did not pass the FDR test; those were linked to manganese ion binding site. In addition, two single GO terms were found, and these were terms for acetylation and cytosol.
- GGA20 had a single significant cluster for transcription and no significant single GO terms.
- GGA21 had a significant GO cluster for NADP, and the same term can be found as the only single GO term for this microchromosome.
- GGA23: six significant annotation clusters passed the FDR test. These included mRNA splicing, ribonucleoprotein, gene silencing by miRNA, translation regulation, stem cell self-renewal protein Piwi, and single-stranded RNA binding. Thirty-five single GO terms were also found that embraced the same functionalities plus the nucleoplasm and poly(A) RNA binding.
- GGA24: out of five clusters, four passed the FDR test. These code for cytoplasmic topological domain, extracellular topological domain, immunoglobin domain, anchored component of membrane and potassium ion import. Seventeen more single GO terms were also found in this microchromosome.
- GGA26 possessed a single significant cluster for BTB/POZ fold, potassium channel ion, and transmembrane transport. We also found 20 single GO terms all linked to cellular transport.
- GGA27 showed one significant annotation cluster for transcription, DNA-binding, homeobox, developmental protein, and embryonic skeletal system morphogenesis. The functional annotation chart displayed 20 single GO terms with the same functionalities and, additionally, nucleus-related one.

**Table 1:** Summary of the msHSBs present in separate chromosomes and groups of chromosomes, and their GO

Chromosome	No. of msHSBs	No. of filtered human orthologs	No. of significant annotated clusters^1^	No. of significant annotated clusters^1^, FDR < 0.05	Clustered GO terms	No. of single GO terms^2^	Single GO terms
**1**	132	876	See below the combined data for 10 macrochromosomes				
**2**	68	579					
**3**	54	403					
**4**	67	397					
**5**	37	355					
**6**	15	174					
**7**	16	230					
**8**	22	181					
**9**	19	176					
**10**	10	165					
**11**	12	107	1	0	cell cycle, tumor suppressor	0	–
**12**	14	115	1	0	neurotransmitter transport	0	–
**13**	13	148	3	1	neuroactive ligand-receptor interaction, postsynaptic cell membrane, ion transmembrane transport, synapse	19	protein binding, neuroactive ligand-receptor interaction, postsynaptic cell membrane, transmembrane transport, synapse
**14**	15	93	0	0	–	2	phosphatase binding, interaction with TP53
**15**	11	150	1	1	TPR repeat	7	acetylation, protein complex, RING, TPR repeat, polysome
**17**	11	129	1	1	metal-binding, DNA-templated regulation of transcription	2	metal-binding, DNA-templated regulation of transcription
**18**	11	57	0	0	–	0	–
**19**	9	101	2	0	manganese ion-binding site	2	acetylation, cytosol
**20**	11	65	1	1	transcription	0	–
**21**	10	46	1	0	NADP	1	NADP
**22**	3	18	0	0	–	0	–
**23**	8	40	6	6	mRNA splicing, ribonucleoprotein, gene silencing by miRNA, translation regulation, stem cell self-renewal protein Piwi, single-stranded RNA binding	35	nucleoplasm, poly(A) RNA binding, mRNA splicing, ribonucleoprotein, gene silencing by miRNA, translation regulation, stem cell self-renewal protein Piwi, single-stranded RNA binding
**24**	10	49	5	4	cytoplasmic topological domain, extracellular topological domain, immunoglobulin domain, anchored component of membrane, potassium ion import	17	cytoplasmic topological domain, extracellular topological domain, immunoglobulin domain, anchored component of membrane, potassium ion import
**26**	5	21	1	1	BTB/POZ fold, potassium channel, ion transmembrane transport	20	BTB/POZ fold, potassium channel, ion transmembrane transport, protein homooligomerization, axon terminus
**27**	3	19	1	1	transcription, DNA-binding, homeobox, developmental protein, embryonic skeletal system morphogenesis	20	nucleus, transcription, DNA-binding, homeobox, developmental protein, embryonic skeletal system morphogenesis
**28**	2	21	0	0	–	0	–
**Z**	42	260	2	1	WD repeat	6	signal transduction, WD repeat
**Macrochromosomes 1–10** **(10 chromosomes)**	440	3536^3^	–	–	–	71	phosphoprotein, membrane, cytoplasm, acetylation, cytosol, metal-binding, transport, transcription, nucleotide-binding, transferase, Ubl conjugation, ATP-binding, isopeptide bond, developmental protein, kinase, cell junction
**Macrochromosomes 1–10 + Z** **(11 chromosomes)**	482	3795^3^	–	–	–	79	phosphoprotein, membrane, cytoplasm, acetylation, cytosol, metal-binding, transport, transcription, nucleotide-binding, transferase, Ubl conjugation, ATP-binding, isopeptide bond, developmental protein, kinase, cell junction, protein transport
**Microchromosomes 11–28** **(16 chromosomes)**	148	1179	10	6	nucleus, transcription, BTB/POZ domain, mRNA splicing, neurotransmitter transport, RNA-binding, metal-binding	39	protein binding, phosphoprotein, nucleus, cytoplasm, acetylation, cytosol, metal-binding, nucleoplasm, transport, membrane, transcription, methylation, RNA-binding
**Microchromosomes 11–19** **(8 chromosomes)**	96	900	6	3	metal-binding, LIM domain, ubiquitin protein ligase activity	13	protein binding, phosphoprotein, cytoplasm, acetylation, cytosol, metal-binding, membrane, methylation, protein transport, ubiquitin protein ligase activity, LIM domain
**Microchromosomes 20–28** **(8 chromosomes)**	52	279	9	7	nucleus, transcription, mRNA splicing, ion channel, potassium transport, cell junction, synapse, embryonic skeletal system morphogenesis, gene silencing by miRNA, axon terminus	37	phosphoprotein, nucleus, acetylation, transcription, transport, poly(A) RNA binding, identical protein binding, cell junction, ion channel, mRNA splicing, synapse

Notes: msHSBs, multi-species homologous synteny blocks; FDR, false discovery rate; GO, gene ontology; ^1^ Annotated clusters that have enrichment score > 1.3. ^2^ Significant single GO terms, FDR < 0.05. ^3^ The online DAVID tool does not compute annotated clusters if a gene list has more than 3000 entries

### EBRs

Using the Evolution Highway genome browser (Fig. 2c), a total of 21 EBRs were detected out of which 13 were also present in the genome of the anole lizard, i.e., being common for all saurian descendants, while eight were specific to avian lineages (Table 2). EBRs on chromosomes 4–6, 8, 9,14, and 18 were either specific to avian lineages or shared between avian lineages and lizards, i.e., being common for all saurian descendants. The identified EBRs made up for 2,401,536 bp, which was approximately 1/500 of the genome. In this total length, 12 filtered genes were found, giving the gene density of five genes per 1 Mb in EBRs. After LiftOver conversions, a list of 19 EBRs was obtained. None of the EBRs contained a significant GO term or a significant GO cluster in our hands.

**Table 2:** Summary of the shared EBRs

EBR No.	Chromosome	Start, bp	End, bp	Length, bp
1^a^	1	8,882,547	8,889,065	6,518
2^a^	1	72,413,370	72,415,249	1,879
3^b^	3	2,394,598	2,406,625	12,027
4^b^	3	5,597,364	5,606,885	9,521
5^a^	3	11,579,794	11,585,862	6,068
6^b^	4	1,871,562	1,872,588	1,026
7^b^	4	19,197,411	19,231,456	34,045
8^b^	5	5,678,440	5,834,079	155,639
9^b^	5	6,517,980	6,533,261	15,281
10^a^	6	6,069,207	6,074,379	5,172
11^a^	6	8,642,096	8,814,146	172,050
12^a^	6	10,015,618	10,021,973	6,355
13^a^	6	10,643,477	10,727,907	84,430
14^b^	6	11,568,835	11,578,014	9,179
15^b^	7	7,336,101	7,339,690	3,589
16^b^	8	9,981,443	9,998,441	16,998
17^a^	9	2,892,593	2,958,453	65,860
18^b^	14	13,596,312	13,672,177	75,865
19^b^	14	14,294,034	14,305,879	11,845
20^b^	18	5,035,833	5,038,416	2,583
21^b^	18	10,250,901	10,252,198	1,297

a, EBRs in avian lineages only (i.e., shared between all bird species studied); b, EBRs shared between lizard and birds.

## Discussion

In the present study, we re-assessed the in-silico data that we previously used to produce the reconstruction of the general avian genome structure, organization and evolution (Romanov et al. 2014b). The six particular birds used here for comparisons with the previous study by Romanov et al. (2014b) were selected because their genomes were sequenced, assembled and annotated at high quality level, with the chicken genome sequence being the standard avian reference genome widely used in comparative genomics. Moreover, these avian species represent major evolutionary lineages of birds, including the orders Galliformes (chicken, turkey) and Anseriformes (duck), which form the basal avian clade Galloanserae of the Neognathae infraclass. The ostrich belongs to the sister, and more ancient, taxon Palaeognathae. Finally, the other two selected species are members of the Neognathae infraclass, Neoaves, represented by the orders Passeriformes (zebra finch) and Psittaciformes (budgerigar), both forming the most recent evolutionary clade Psittacopasserae, remarkable for including species with vocal learning. The study of msHSBs, EBRs, and their related gene ontology as described in our previous (Romanov et al. 2014b) and current investigations has essentially benefited from the coverage of major avian evolutionary lineages. We took into account that we now have better genome assemblies and alignments as well as improved GO analysis components and new bioinformatic tools and algorithms. To this end, we employed the powerful inhouse computing pipeline (O’Connor et al. 2018a; Abdelmanova et al. 2021) that encompassed Evolution Highway (for defining msHSBs and EBRs), LiftOver, BioMart, and DAVID. We inferred useful information from the msHSBs and EBRs in achieving our final aim, which was the functional characterization of these evolutionarily conserved elements in the avian genome using GO terms. For this purpose, we did not analyze directly chicken genes located within the msHSBs and EBRs and retrieved orthologous human genes for these chicken genome regions using BioMart in Ensembl, a key tool in the pipeline of our overall analysis. Rather, we used human orthologs instead of chicken genes because human genes are much better functionally annotated than chicken genes (O’Connor et al. 2018a). As a result, we were able to find some specific and significant gene enrichments and the appropriate GO terms for the genomic regions in birds and their ancestors (including extinct dinosaurs) that correspond to msHSBs and EBRs.

Having at hand the galGal4 assembly and DAVID Knowledgebase 6.7, Romanov et al. (2014b) stated that limited evidence exists to support the concept that a clustering of genes with related functions on the same chromosome may be one explanation for the hypothesis that microchromosomes reflect highly conserved blocks of interchromosomal synteny. Unlike the GO estimation by Romanov et al. (2014b), we used the updated chicken reference genome sequence (galGal6) and the updated DAVID GO database (Knowledgebase 6.8) to discover msHSBs on multiple microchromosomes, characterize them by specific GO terms, and infer eventually the meaningful GO terms and clusters. Romanov et al. (2014b) only found this on GGA16 which was linked to the immune system. Here, however, we established that many association between HSB located on specific avian chromosomes and specific GO functions, including msHSBs on GGA13 that were specific to neurons, those on GGA23 to RNA, those on GGA26 to cellular transport, and those on GGA27 were linked to embryonic development, among many others.

As the msHSBs made a total 807,632,434 bp in about 1.2 Gb of the reference sequence, we concluded that throughout the evolution of avian species ~ 2/3 of their genome was conserved. We found that microchromosomal msHSBs were around twice as gene dense as those on macrochromosomes. This was highly consistent with other similar estimates of overall gene density on microchromosomes relative to macrochromosomes (e.g., Smith et al. 2000; Abdelmanova et al. 2021), proving a clear negative relationship between gene density and chromosome type by length in the avian genome (International Chicken Genome Sequencing Consortium 2004).

While our msHSB-derived GO results for the six birds were provided for individual chromosomes or chromosome groups (Table 1), Farré et al. (2016) used a different approach by unveiling the most common signatures of gene-functional enrichment for all pooled macro- and microchromosomal msHSBs. Nevertheless, few GO terms revealed for embryonic morphogenesis, nucleotide binding, and transcription were shared between the two studies. Damas et al. (2018) searched for msHSB-specific GO terms present on few reconstructed avian ancestral microchromosomes. Among GO terms enriched on those chromosomes, there were those identified for microchromosomes in the present study and relevant to binding, transcription, membrane, extracellular topological domain (region), protein binding, and substrate-specific channel activity.

In our observations, EBRs were rarer genomic features than HSBs in multispecies comparisons. Only 21 EBRs were found in the comparisons of six avian genomes and that of the anole lizard (Table 2). Moreover, 13 of these EBRs are also shared with lizards, meaning they have been conserved since the common ancestor of birds and lizards, which would be the saurian ancestor (Maddison and Schulz 2007). The eight other EBRs were specific to avian lineages and were therefore considered less ancient than the 13 EBRs coming from the saurian ancestor.

In terms of gene density, these EBRs had five filtered genes per 1 Mb, while msHSBs had the overall gene density of 9.8 genes per 1 Mb. Since the estimate for EBRs-specific gene richness was unlikely to be significant, we cannot deduce that msHSBs and EBRs were characterized by unsimilar gene density. Accordingly, the fact that we found no significant GO terms for the genes in EBRs does not mean they bear no common functionality, unlike the genes in msHSBs.

## Conclusions and future directions

In the course of this investigation, we employed bioinformatic tools that enabled us to expand our knowledge about the structural and functional organization of the genome in birds and their ancestors. For this purpose, we improved the whole pipeline algorithm for analyzing the genomic datasets by tuning the pipeline settings (O’Connor et al. 2018a) and using updates for chicken reference sequence as well as BioMart and DAVID databases. Importantly, Evolution Highway-, LiftOver-, BioMart- and DAVID-based analyses enabled to retrieve and examine 630 msHSBs using the genomes of six bird species. We succeeded in identifying meaningful functionalities within microchromosomal and macrochromosomal genomic regions of interest, unlike our previous work (Romanov et al. 2014) when we were unable to show significant gene ontology for most chromosomes or chromosome groups such as macrochromosomes vs. microchromosomes. Our findings for msHSBs also supported the previous estimates that microchromosomes have twice as many genes per 1 Mb as macrochromosomes.

Overall, based on the presented *in silico* analyses, we can conclude that msHSBs are gene rich regions of bird genomes that are kept together in the avian evolution and bear specific gene ontologies. EBRs represent, to a larger extent, more ancient genomic features dating back to the saurian ancestor and, therefore, are subjected to reuse. They are also rich in terms of gene content, although we were unable to characterize them by any significant functionality (due to a smaller number of EBRs observed and usable to identify significant GO terms).

In the future, it would be important to increase our knowledge about the evolution and temporal stability of msHSBs by increasing the number of avian genomes and outgroups. This could provide information about msHSBs that are characteristic of specific avian lineages and others that may be shared across avian lineages, showing more ancestral origins. In addition, it would be interesting to examine each avian macrochromosome separately to uncover their individual msHSBs and GO features. Finally, studying HSBs shared between birds and lizards could potentially help documenting whether the functional specificity of the genes present on these microchromosomes is specific to avian lineages or is shared with the saurian descendants.

## Data Availability

The datasets generated during and/or analyzed during the current study are available from the corresponding author on reasonable request.
